# Identification of novel genetic factors underlying the host-pathogen interaction between barley (*Hordeum vulgare* L.) and powdery mildew (*Blumeria graminis* f. sp. *hordei*)

**DOI:** 10.1371/journal.pone.0235565

**Published:** 2020-07-02

**Authors:** Maria Pogoda, Fang Liu, Dimitar Douchkov, Armin Djamei, Jochen C. Reif, Patrick Schweizer, Albert W. Schulthess

**Affiliations:** Department of Breeding Research, Leibniz Institute of Plant Genetics and Crop Plant Research, Gatersleben, Germany; Institute of Genetics and Developmental Biology Chinese Academy of Sciences, CHINA

## Abstract

Powdery mildew is an important foliar disease of barley (*Hordeum vulgare* L.) caused by the biotrophic fungus *Blumeria graminis* f. sp. *hordei* (*Bgh*). The understanding of the resistance mechanism is essential for future resistance breeding. In particular, the identification of race-nonspecific resistance genes is important because of their regarded durability and broad-spectrum activity. We assessed the severity of powdery mildew infection on detached seedling leaves of 267 barley accessions using two poly-virulent isolates and performed a genome-wide association study exploiting 201 of these accessions. Two-hundred and fourteen markers, located on six barley chromosomes are associated with potential race-nonspecific *Bgh* resistance or susceptibility. Initial steps for the functional validation of four promising candidates were performed based on phenotype and transcription data. Specific candidate alleles were analyzed via transient gene silencing as well as transient overexpression. Microarray data of the four selected candidates indicate differential regulation of the transcription in response to *Bgh* infection. Based on our results, all four candidate genes seem to be involved in the responses to powdery mildew attack. In particular, the transient overexpression of specific alleles of two candidate genes, a potential arabinogalactan protein and the barley homolog of *Arabidopsis thaliana’s* Light-Response Bric-a-Brac/-Tramtrack/-Broad Complex/-POxvirus and Zinc finger (*AtLRB1*) or *AtLRB2*, were top candidates of novel powdery mildew susceptibility genes.

## Introduction

Crop protection is mostly provided by the application of chemical agents, which can be harmful to the environment while a more sustainable disease control is resistance breeding [1,2]. Nonetheless, this often requires extensive field-testing of plant material, due to the influence of the environment on plant diseases. Considering this, characterizing plant resistance mechanisms provides important hints for selection and thus assists breeding [1,3,4].

One relevant foliar disease of barley (*Hordeum vulgare* L. subsp. *vulgare*) and wheat (*Triticum aestivum* L.) is powdery mildew (PM), which is caused by the biotrophic fungi *Blumeria graminis* f. sp. *hordei* (*Bgh*) and *Blumeria graminis* f. sp. *tritici* (*Bgt*), respectively [5,6]. Both fungi grow usually only on the corresponding host [7] while infection with either *Bgt* or *Bgh* can lead to severe yield losses of wheat and barley [8,9], which are the second and fourth most produced grains in the world, respectively [10]. Moreover, barley is a model organism to study plant-pathogen interactions in cereals. For instance, important resistance loci like *Mla*, *MlLa*, and *Mlg* [11,12], as well as susceptibility factors like *Mlo* [13–15] have been identified in barley and some of them are even relevant for non-cereal species [13,16,17]. Resistance can be classified into: a) race-specific, which is active against a particular pathogen species (race/isolate); and b) race-nonspecific [18,19]. The resistance provided by race-nonspecific resistance genes is mostly independent of the pathogen race/isolate, the time of its onset, and the plant developmental stage or the environment [18–20]. The use of monogenic resistance genes provides usually strong race-specific protection but results often in an early breakdown of the resistance caused by the co-evolution of host and pathogen. These ‘boom-and-bust’ cycles occur particularly if the same resistant cultivar is grown on a large area across some years [4]. One exception to this rule is the race-nonspecific PM resistance conferred by loss-of-function mutations in the *Mlo* gene, which was durable during several decades [13–15]. This gene represents a crucial susceptibility factor necessary for fungal development and virulence [13]. The underlying mechanism of the recessive *mlo* resistance is related to basal defense reactions, which are of special interest for plant breeding due to their regarded durability and broad-spectrum activity [13,17,21]. Nonetheless, despite the promising potential of race-nonspecific resistance, its use is quite limited in breeding. This is presumably due to a lack of general understanding of race-nonspecific mechanisms, which are usually of polygenic nature and thus more complicated to handle in breeding [21].

Domestication and high selection intensities have reduced genetic diversity in modern material [22,23] and landraces, as well as wild crop ancestors, may contain valuable genetic variation for agronomical traits awaiting to be mined [24–26]. Genome-wide association (GWA) studies proved to be versatile in the identification of candidate genes or loci in genetically diverse material [27,28]. Particularly, GWA studies have been successfully used to identify PM resistance genes/loci [29–31]. Provided a highly diverse population, the mapping resolution of GWAS depends to a great extent on marker density [32]. In this regard, exome capture delivers highly dense data in a cost-efficient manner and focuses on variations in protein-encoding regions [33–35]. For most breeding applications a significantly associated marker could be enough [36], but for the understanding of a resistance mechanism or its regulation, it is necessary to identify the corresponding causal gene and its function *in vivo*. Despite its importance, the functional validation of candidates identified by GWA studies is often not provided [37,38]. In particular, in the barley-*Bgh* pathosystem, transient assays are an established tool for the initial validation of identified candidates [39]. Analyses of race-specific PM loci revealed strong allelic effects indicating the importance of the exploitation of this diversity [40,41]. In contrast, knowledge of allele specificities of race-nonspecific resistance genes is quite limited [31] and in particular, the functional validation of alleles in different genetic backgrounds is usually missing.

We started our approach by evaluating the potential race-nonspecific seedling resistance or susceptibility of 267 barley genotypes based on detached leaves against two poly-virulent *Bgh* isolates. Further, we selected 201 genotypes to create a collection, which represents a broad proportion of the genetic diversity existent in barley and evaluated its suitability to conduct a GWA study. We fitted a mixed linear model for 424,567 single nucleotide polymorphisms (SNPs) to identify potentially important factors associated with race-nonspecific, quantitative plant responses against PM. In particular, we began to validate and analyze specifically allelic effects to gain first insights into the functionality of four promising candidate genes underlying two association peaks on chromosome 5H that co-localize with the seedling PM resistance locus *Rbgq15* [11].

## Material and methods

### Plant material

Based on an initial characterization of the resistance against the PM isolate D35/3 (JKI-242) of 459 genotypes of the barley Whealbi (EU FP7 n°FP7- 613556) collection, a representative panel of 267 genotypes was chosen (hereafter, phenotyping-panel), spanning the complete range of PM susceptibility. Passport data of the phenotyping-panel (**S1 Table**) were obtained from the official germplasm description for the barley Whealbi collection (https://wheat-urgi.versailles.inra.fr/Projects/Achieved-projects/Whealbi) and were complemented with data from Bustos-Korts et al. [42]. The susceptible cultivar Morex and the moderately susceptible cultivar Roland were included as positive controls for infection efficiency during the experiments.

Leaf segments of JB Flavour and Roland were used for the weekly maintenance of the *Blumeria graminis* (DC.) E. O. Speer f. sp. *hordei* (*Bgh*) isolates D35/3 (JKI-242) and RiIII (JKI-75), respectively. The plants were grown for 7d under long-day conditions (16h) at 20°C in plant growth chambers. A mixture of Maythorpe:Ingrid:Golden-Promise (2:2:1) cultivars grown for 6 d in a glasshouse chamber under long-day conditions at 22°C/20°C, was used for the maintenance of the isolate CH4.8 (JKI-247). Infected plants and inoculated material were incubated at 20°C in plant growth chambers at long-day conditions.

### Powdery mildew resistance screening

The barley genotypes of the phenotyping-panel were grown in 9x6 multi-pot trays filled with compost soil from the IPK nursery, without fertilization. They were grown for 12d in a glasshouse chamber under long-day conditions at 20°C/16°C (day/night temperature). The PM isolates D35/3 and RiIII were selected regarding their poly-virulent nature to a smaller set of 33 barley genotypes known for their differential responses against PM (**S2 Table**). A detached leaf assay was conducted for the resistance scoring, in which second leaves of seedlings were cut and placed on standard 4-well multi-titter plates filled with 1% Phytoagar solution supplemented with benzimidazole (20 mg/l). In this essay, leaves were inoculated with fresh PM spores (8-10 conidia/mm^2^) and disease symptoms were estimated (scored by eye) 7d after inoculation [31,43]. To reveal the quantitative characteristic of the PM infection, the infected leaf area was scored as the estimated percentage of leaf area covered by macroscopically visible disease symptoms. In this sense, a decreased percentage of infected leaf area is interpreted as enhanced quantitative resistance while higher percentage values correspond to increased quantitative susceptibility. We introduce here the term “experiment” to describe all those individual plants that were grown and inoculated simultaneously. In each experiment and for each isolate, five different plants (technical replicates) were inoculated per tested genotype while each genotype of the phenotyping-panel was assessed in at least three different experiments (experimental replicates) per isolate.

### Exome capture data

The exomes of a total of 403 genotypes of the barley Whealbi collection were genotyped by a hybridization-based sequence capture platform [42]. SNP data were then subtracted for the overlapping accessions between the Whealbi collection and our phenotyping-panel. The final set of 424,567 SNPs was generated by removing SNPs with a minor allele frequency <5% leading to an average SNP density of 6.9 SNP/kb.

### Processing of phenotypic data

To determine the association between the two isolates, the arithmetic means per genotype per isolate were calculated from the values normalized with the control cultivar Morex after outlier test correction (ROUT Q = 1%, performed with GraphPad [44] Prism version 7.01 for Windows). The significance of Pearson correlation coefficients between the disease scores of D35/3 and RiIII was assessed by a two-tailed t-test. After removing 21 completely resistant genotypes coming from the phenotyping panel (**S1 Table**, see Results for further details), 201 genotypes having SNP and phenotypic data were retained for further analyses. Here onwards these 201 genotypes are referred to as the GWA-panel.

Linear mixed models [45,46] are useful for estimating genotype performance from non-orthogonal phenotypic data [47,48]. We used a two-step mixed model approach to obtain the best linear unbiased estimations (BLUE) of genotypes from the GWA-panel. In the first step, the following linear mixed model was separately fitted to the data of each experiment:

$$y_{\left(i\right)jk}=\mu +g_{j}+p_{k}+e_{\left(i\right)jk}$$ (1)

where *y*_*(i)jk*_ is the non-normalized detached leaf assay value for the *j*th genotype evaluated within the *k*th plate in the *i*th experiment, μ corresponds to the common mean of the material, *g*_*j*_ is the effect of *j*th genotype, *p*_*k*_ denotes the effect of the *k*th plate, and *e*_*(i)jk*_ is the residual variation. The common mean and genotypic effects were assumed as fixed factors, while the rest of the factors were considered random. In a second step, the genotype BLUEs from each experiment were analyzed together using the model:

$$y_{ij}=\mu +\xi_{i}+g_{j}+e_{ij}$$ (2)

where *y*_*ij*_ is the BLUE of fungal infection for the *j*th genotype evaluated in the *i*th experiment, μ denotes the common mean across experiments, *g*_*j*_ corresponds to the effect of the *j*th genotype across experiments, ξ_*i*_ indicates the effect of the *i*th experiment and *e*_*ij*_ is the residual variation. Common mean and genotypic effects were considered fixed, while the rest of the factors were assumed as random. Furthermore, genotypic variances (σ_*g*_^*2*^) were estimated for each experiment and across experiments using Eqs (1) and (2) but considering genotypes as random. In parallel, error variances ($\bar{v}$) for each experiment and across experiments were computed as the squared mean standard error of the difference between two genotypes according to Eqs (1) and (2), respectively. Variance estimates were subsequently used to compute the repeatability of each experiment and isolate as well as the heritability for each isolate according to Piepho and Möhring [49]:

$${\bar{H}}^{2}=\frac{\sigma_{g}^{2}}{\sigma_{g}^{2}+\bar{v}/2}$$ (3)

Based on the calculated BLUEs, an artificial maximal infection (*Max*) was generated *in silico* for each genotype of the GWA-panel by selecting the higher infection value of the two isolates.

### Population structure and linkage disequilibrium

Pairwise Rogers’ distances (RDs) [50] were estimated among genotypes using SNP data of the GWA-panel and were subjected to hierarchical cluster analysis for studying the population structure. Further, the Pearson correlation coefficients of the RD and the phenotypic distance matrices from the genotypes of the GWA-panel were assessed by a Mantel test implemented in R [51] Version 3.5.1.

The pairwise linkage disequilibrium (LD) as *r*^*2*^ of the significant SNPs of the selected candidate genes was determined by TASSEL5 [52] using 50 SNPs as sliding window size and excluding heterozygous SNPs.

### Genome-wide association study

A standard linear mixed model [53] was fitted at each SNP position on the plant responses of D35/3 and RiIII as well as their maximal infection (*Max*):

y=Xβ+Zg+e (4)

where *y* is the vector containing the BLUEs across experiments of the genotypes of the GWA-panel, β denotes a vector of fixed effects containing the common population mean, the effects of the subpopulations portrayed by the RD and the allele substitution effect of the tested marker, *g* corresponds to a vector containing the random genetic background effects of the genotypes in the GWA-panel, *X* and *Z* represent design matrices that relate *y* to β and *g*, respectively, while *e* is a vector of random errors. Within the *X* matrix, those columns corresponding to subpopulations are the memberships of genotypes to them. Vectors *g* and *e* were assumed as *g~N*(0,σ_*g*_^2^*K*), *e~N*(0,σ_e_^2^*I*), where *K* is a marker-derived kinship matrix calculated as 2*(1-RD) [54], *I* is an identity matrix, while σ_*g*_^2^ and σ_e_^2^ are the corresponding variances. The genome-wide multiple-test Type I error was controlled by using the effective number of markers (Meff_G) necessary to explain 99.8% of the chromosome-wise calculated LD matrices as proposed by Gao et al. [55]. By dividing the nominal Type I error rate (α_e_) of 0.05 by Meff_G = 1,414 a significance threshold of -log10(*P*) = 4.45 was obtained. Mixed model Eqs (1), (2) and (4) were fitted using ASReml-R [56] in R environment [57] Version 2.15.

At least 2kb of the flanking genomic regions surrounding the significant SNPs of the *Max* trait from the barley reference genome [34] were extracted via the IPK Galaxy Server and used for a ‘BLASTN’ analysis with the IPK BLAST Server [58] and the NCBI server. Details about the used databases are provided in the **S3 Table**.

### Characterization and functional validation of the selected candidate genes

Significant SNPs for the *Max* trait located within annotated gene models [34] were curated by comparison of the gene models with homologous sequences identified in the related species *Aegilops tauschii* subsp. *tauschii* and *Brachypodium distachyon*. Combinations of the selected SNPs within the curated gene model defined the corresponding alleles. Therefore, the term allele in this context corresponds to an SNP haplotype. Means of the infected leaf area of all genotypes carrying the respective alleles were calculated and the significance of the difference between the corresponding alleles was assessed via a two-tailed t-test. Linear models were fitted in R environment [51] Version 3.5.1 to determine the proportion of the variation that is explained by the candidate genes.

The closest protein homologs of the candidate genes were identified via BLAST analyses. In case of the related species *A*. *tauschii* and *B*. *distachyon*, the protein sequences of the NCBI BLAST hits were selected, and the homologs of *Arabidopsis thaliana* and rice (*Oryza sativa* ssp. *japonica* cv. *Nipponbare*) were identified via ‘BLASTP’ against the TAIR server [59] and the rice genome annotation project server [60], respectively. For candidate gene CG-4, the protein sequences described by Kimura et al. [61] were chosen. The off-targets for RNAi predicted by the si-Fi21 tool [62] were assumed as candidate homologs of barley. The obtained sequences per candidate were aligned with the online tool ‘Clustal Omega’ [63] (**S1A-S4A Figs in S1 File**) and domains were predicted with the ‘Pfam 32.0 sequence search’ [64] and the ‘NLS mapper’ [65] for candidate CG_3 using default settings. The nucleotide sequences of the defined candidate alleles were generated based on the annotated SNPs and the obtained sequencing results of the cloned alleles. These sequences were translated into putative proteins with the ‘ExPASy -Translate tool’ [66]. The generated protein sequences of CG_4 were analyzed with the ‘SignalP 5.0 tool’ [67].

The expression of candidate genes was analyzed in Microarray data published by Delventhal et al. [68] based on the SCRI_Hv35_44k_v1 chip (Agilent_20599). The corresponding U35 sequence identifier and probe-IDs of the candidates were identified via BLAST against the HarvEST barley database (**S3 Table**). The significance of the difference in gene expression was assessed from three biological replicates via unpaired two-tailed t-test between inoculated and non-inoculated samples per time point.

### Particle bombardment

For the Transient-Induced Gene Silencing (TIGS), hairpin constructs were created from cDNA fragments of 190-500bp length amplified with the Phusion High-Fidelity DNA Polymerase (Thermo Fisher Scientific, Waltham, USA). For this purpose, total RNA was extracted from 7d old leaves of the cultivar Morex using the RNeasy® Plant Mini Kit (Qiagen, Hilden, Germany) and subsequently treated with DNase I (Thermo Fisher Scientific). Afterwards, cDNA was generated using the RevertAid M-MuLV RT Kit and Oligo(dT)_18_ primer (Thermo Fisher Scientific) following the manufacturer’s instructions. The candidate primers (**S4 Table**) were designed based on the prediction of the si-Fi21 tool [62] for the candidate regions with the most efficient small interfering RNAs (siRNAs) without predicted off-targets. The corresponding primer positions were indicated in the gene models in **S1B-S4B Figs in S1 File**. Cloning and particle bombardment was performed similarly to the method described by Douchkov et al. [69]. After 3 d, the leaf segments of cultivar Morex and WB-052 (HOR2573) were inoculated with the PM isolate CH4.8 (spore density: 450–530 conidia/mm^2^; **S2 Table**). To determine the susceptibility index (SI), the proportion of the GUS-stained cells with growing haustorium to the total number of transformed (GUS-stained) cells was calculated. Then, the relative SI (proportion of the SI of the test gene to the SI of the empty vector pIPKTA30N) was calculated. The significance of the difference was assessed from five independent experiments via one-sample two-tailed t-test from log_2_-transformed values against the hypothetical value ‘0’.

For the overexpression constructs, the full-length candidate gene sequence was amplified from genomic DNA extracted with the DNeasy® Plant Mini Kit (Qiagen) according to the manufacturer´s instructions. The amplification was performed with the Phusion High-Fidelity DNA Polymerase (Thermo Fisher Scientific). Primer details were provided in the **S4 Table** and **S1B-S4B Figs in S1 File**. The different alleles were amplified either from the susceptible genotype Morex or the resistant WB-052 and the corresponding fragments were gel-purified with the GeneJET PCR purification Kit (Thermo Fisher Scientific). The blunt-end cloning into pIPKTA9 [70] was performed similarly as described for the TIGS constructs but using *SmaI* (Thermo Fisher Scientific) instead of *SwaI*. The correct orientation of the insert was determined by PCRs and sequencing. The particle bombardment and the microscopical analysis were performed similarly to the TIGS assay, but the PM inoculation was performed after 4h (spore density: 150–250 conidia/mm^2^) and pIPKTA9 served as empty vector control. Each construct was tested in four biological replicates. All work reported in this study was performed following the appropriate biosecurity measures according to the German legislation.

## Results

### The diverse barley material showed high variation for powdery mildew seedling resistance

**Fig 1:**
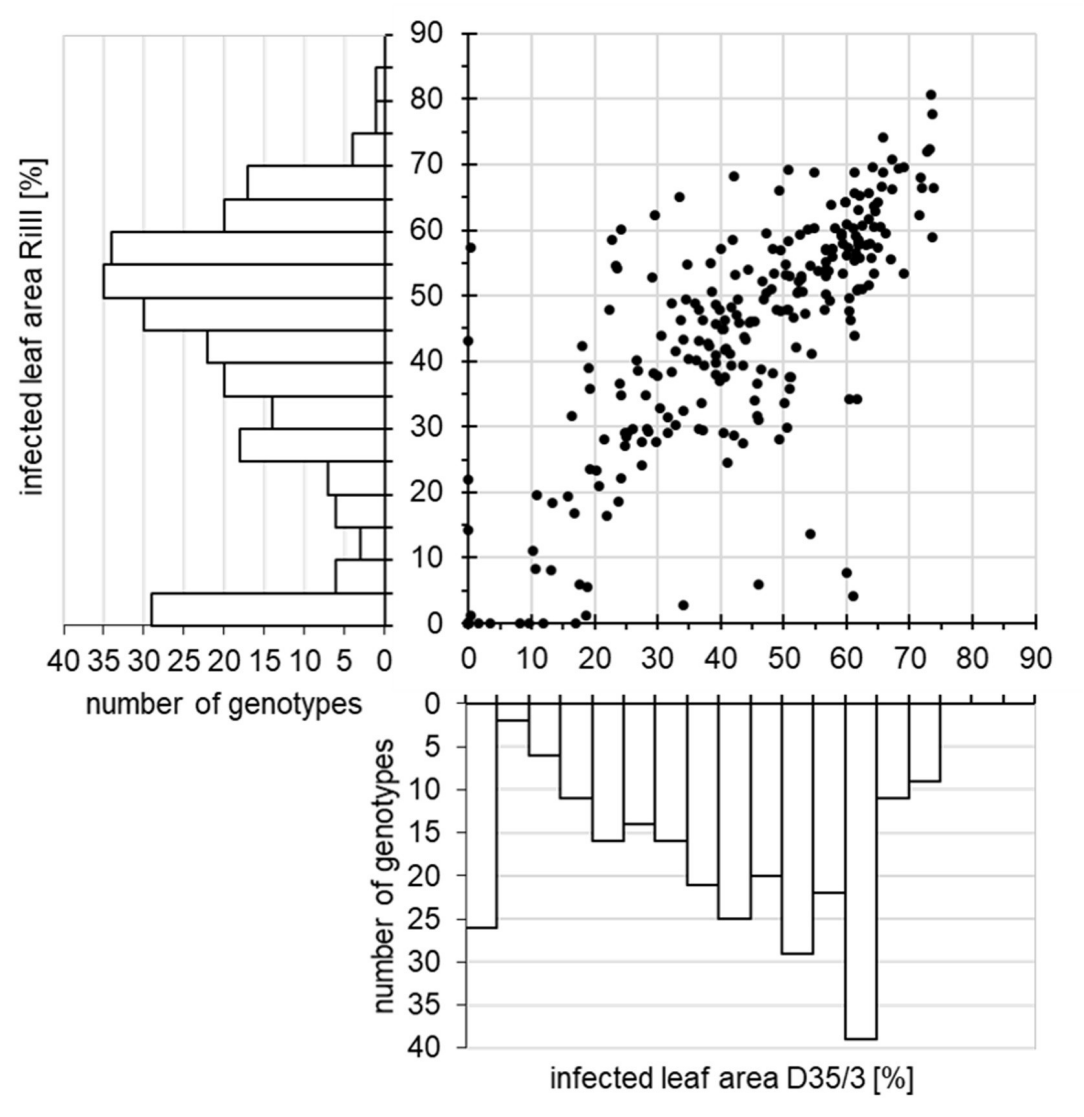
Biplot of the phenotypic distribution of the 267 *Hordeum vulgare* accessions included in the phenotyping-panel. Phenotypic values correspond to the arithmetic mean (n ≥3) of the normalized infected leaf area [in %] for the *Blumeria graminis* f. sp. *hordei* isolates D35/3 vs. RiIII and the corresponding absolute frequencies of genotypes per interval and isolate.

The phenotyping-panel includes spring and winter as well as two- and six-rowed barley types (**S1 Table**). Furthermore, this panel is constituted by modern elite (25%) and traditional material like landraces and old cultivars (70%), as well as by wild barley accessions (5%). To cover key world production regions of barley, the 267 genotypes of the phenotyping-panel were selected mainly from Europe (23%), the Middle-East (22%), Africa (20%), and Asia (17%). In the detached leaf assays, the phenotyping-panel spanned the complete range of susceptibility (**Fig 1**and **S1 Table**) against the two poly-virulent PM isolates, which were virulent against 22 of the most common (European) resistance specificities (**S2 Table**).

Genotype responses against both isolates were strongly correlated (*r* = 0.81, *p-*value <0.0001). The majority of the material of the phenotyping-panel was susceptible to at least one isolate but also 21 completely resistant genotypes against both isolates (arithmetic means of the normalized infected leaf area ≤ 1.5%) were identified (**Fig 1**and **S1 Table**). These genotypes were excluded from further analysis because their phenotype resembled the classical qualitative resistance and the focus of the study was put on the genotypes with a quantitative resistance phenotype. In addition, forty-five genotypes of the phenotyping-panel lack exome capture data [42], leading to the final GWA-panel with 201 genotyped barley genotypes having data on quantitative PM resistance. The high heritability (D35/3: 0.88 and RiIII: 0.87) and reproducibility of the different experiments (D35/3: 0.65-0.95 and RiIII: 0.70-0.93) indicate that the observed phenotypic variation is mostly genetically determined.

### Population structure does not affect the powdery mildew resistance

**Fig 2:**
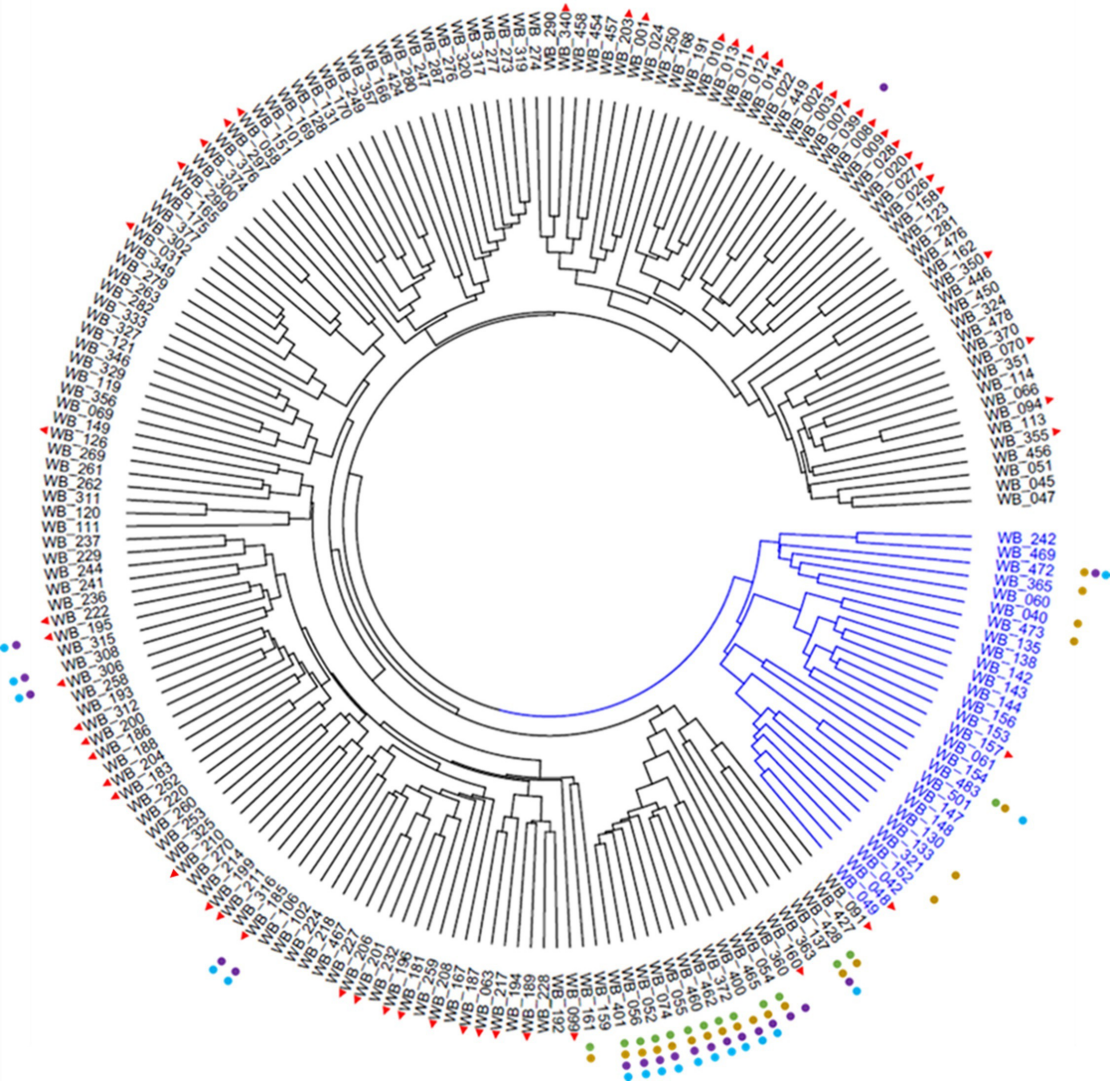
Hierarchical cluster analysis of the 201 *Hordeum vulgare* accessions of the GWA-panel based on the Rogers‘ distance. The identified subpopulation of 28 genotypes is highlighted in blue, modern material is labeled with red arrow-heads and carriers of the resistant alleles are indicated with colored dots: green–CG_1; ochre–CG_2; violet–CG_3; blue–CG_4.

The RDs indicate the presence of two major clusters within the GWA-panel, where 28 genotypes were more closely related to each other than to the rest of the collection forming a small subpopulation (**Fig 2**).

Most of these landraces and wild barleys originate in South East Asia (75%) or the Middle-East (18%). Nevertheless, the subpopulation does not affect PM resistance because the arithmetic means of the infected leaf area of the two clusters (28 genotypes: 46.0 ± 19.9% and 173 genotypes: 49.9 ± 15.3%) are statistically not different from the one of the GWA-panel (49.4 ± 16.0%). Four further sub-clusters were mainly determined by the modern material or by traditional cultivars and landraces of North Africa and mixed origins (**Fig 2**and **S1 Table**). Regarding the low association of RDs and phenotypic distances (r_*Max*_ = 0.12, *p-*value <0.001; r_*RiIII*_ = 0.10, *p-*value <0.002 and r_*D35/3*_ = 0.08, *p-*value <0.001), only marginal effects of the population structure on the association study are expected. Therefore, the sub-clusters were not explicitly modeled during the GWA study.

### Identification of candidate genes based on a genome-wide association study

**Fig 3:**
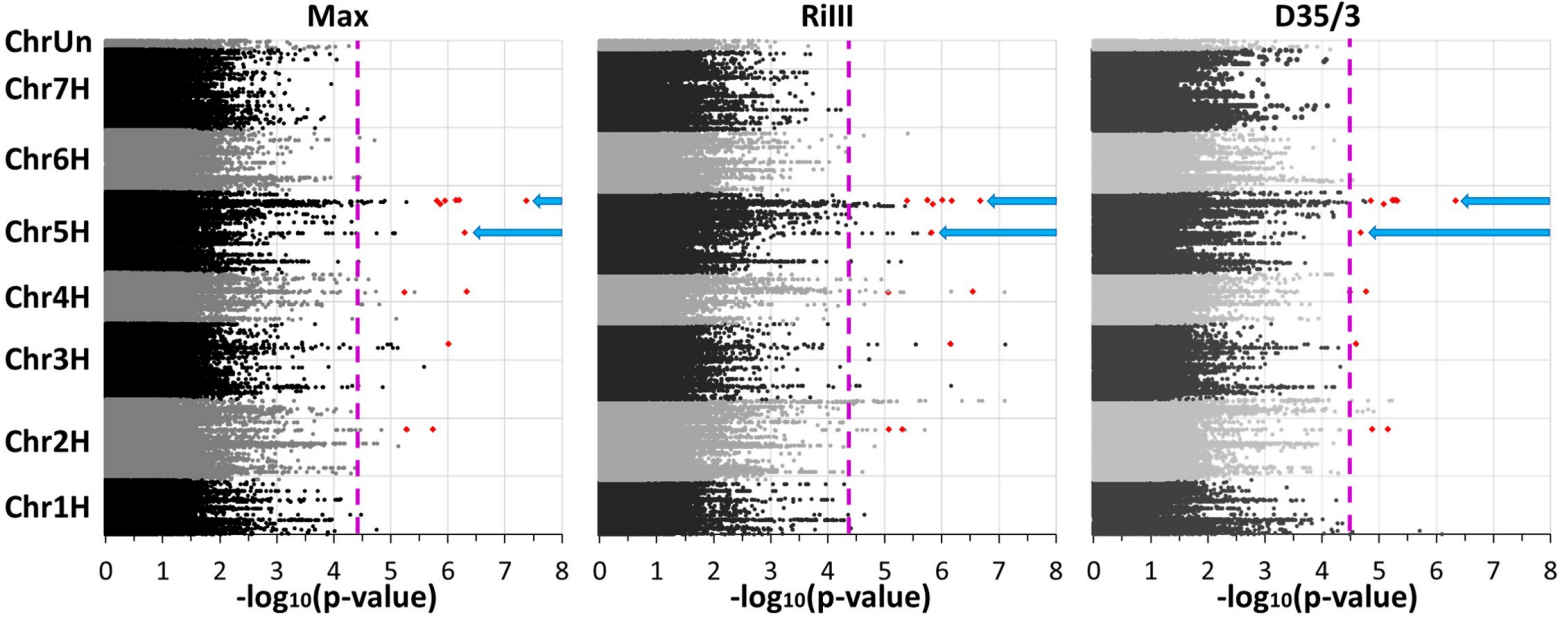
Manhattan plots of the–log_10_-transformed *p*-values for *Max* trait, RiIII, and D35/3 severities. The significance threshold of -log_10_ (*p-*value) *=* 4.45 is depicted as a pink dotted line and the blue arrows represent the peaks from which the four candidate genes have been selected. The 17 SNPs, which were significantly associated with all three traits, are indicated as red diamonds.

In addition to the single isolate resistance scores, an artificial maximal infection (*Max*) was created *in silico*, based on the highest infection value from both isolate sets. This *Max* trait was generated under the assumption that effects on the resistance caused by the different isolates were minimized and that the observed effects represent thus race-nonspecific responses. The linear mixed model in Eq (4) was fitted for the BLUEs of the three traits (RiIII, D35/3, and *Max*) at each of the 424,567 selected SNP positions. Based on the significance threshold of -log_10_ (*p-*value) = 4.45, a total of 214 SNPs located in 15 loci and distributed over six chromosomes, were significantly associated with PM resistance (**Fig 3**and **S5 Table**). Among them, 17 SNPs were in particular significantly associated with all three traits.

Chromosome 7H was the only chromosome without significant associations. Among these associations, five peaks on chromosomes 2H to 5H were simultaneously significant for the three traits while two peaks co-localize with the seedling PM resistance locus *Rbgq15* on chromosome 5H [11]. The involvement of this genomic region in the barley-*Bgh* interaction was further supported by recent results of Hunt et al. and Gupta et al. [71,72]. To further assess this promising region on chromosome 5H (**S5 Fig in S1 File**), the reference genome [34] was used to define candidate genes, whose SNPs were found to be associated with the *Max* trait. Within the first and the second peak, three and twelve candidate genes were identified respectively. The until now identified race-nonspecific resistance genes display various functions [1] and thus, the annotated candidate gene function was not considered as sufficient criterium to select promising candidate genes. Instead, the identified candidate gene models (**S1A-S4A Figs in S1 File**) were verified by comparison to similar sequences of (predicted) genes in related species, full-length cDNA and EST sequences to minimize the risk of the selection of pseudogenes (**S6 Table**). Further, the presence of transcripts in public databases, as a proof that the gene is expressed, and the possibility to design specific primers that amplify these genes from different genetic backgrounds formed also part of the selection criteria. As a result, four candidate genes, denoted as CG_1 to CG_4, respectively, were considered for further analyses (**Table 1**and **S6 Table**).

**Table 1:** Annotated information of the selected candidate genes mapping on chromosome 5H along with their corresponding significantly associated Single Nucleotide Polymorphisms (SNPs).

					-log10(*P*)		
Candidate	Gene-ID^a^	Start^b^	End^b^	SNP^c^	*Max*	RiIII	D35/3
CG_1	HORVU5Hr1	554454779	554457103	SNP554456783chr5H	5.07	5.79	3.46
	G078000						
CG_2	HORVU5Hr1	554737443	554738375	SNP554738172chr5H	6.30	5.82	4.68
	G078330						
CG_3	HORVU5Hr1	649208466	649212390	SNP649211044chr5H	4.83	3.89	4.36
	G116860						
CG_4	HORVU5Hr1	650973514	650980482	SNP650973939chr5H	5.96	5.39	5.32
	G117650			SNP650976700chr5H	7.38	6.68	6.34

^a^ Annotation in Morex [34].

^b^ Physical start and end gene position in the reference genome in base pairs.

^c ^Name of the significant SNP.

### Altered transcript levels after powdery mildew attack

**Fig 4:**
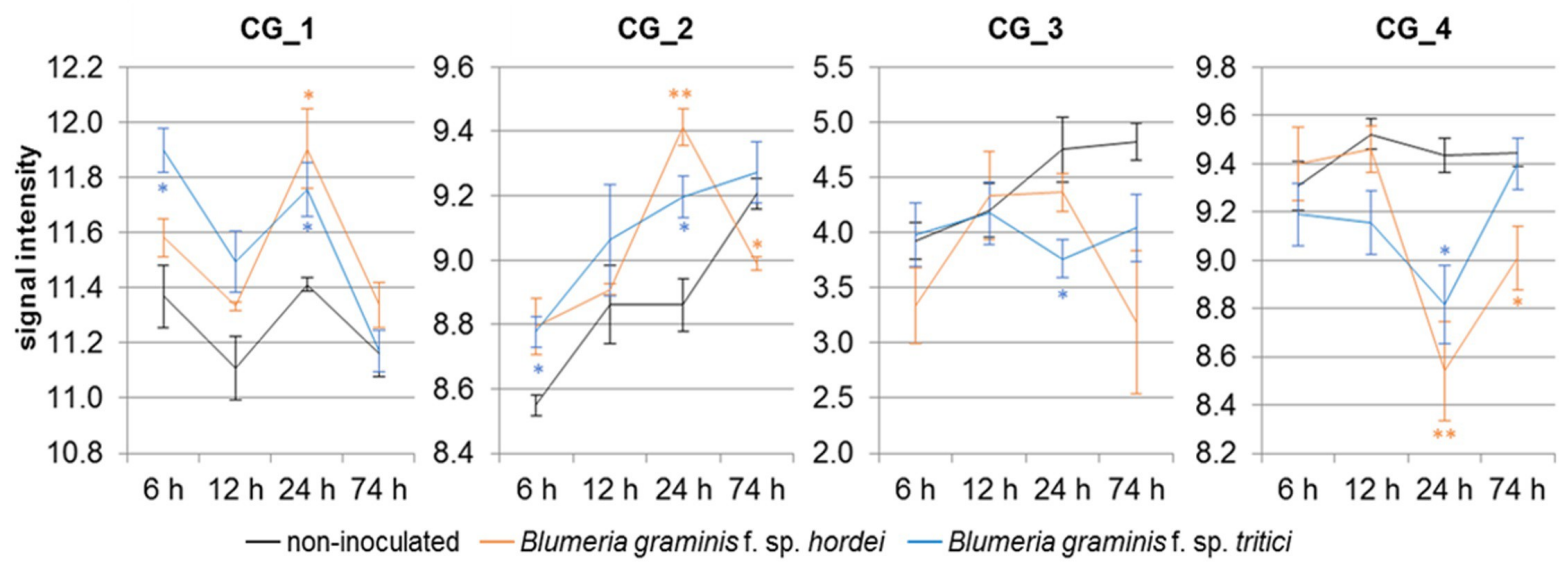
Relative expression of the four Candidate Genes (CG) in powdery mildew attacked epidermis cells. The quantile-normalized signal intensities represent the relative expression of each CG based on Microarray data [68] for non-inoculated, *Blumeria graminis* f. sp. *hordei* (isolate CH4.8) or *Blumeria gramini*s f. sp. *tritici* (isolate FAL 92315) inoculated epidermal peels of the resistant barley cultivar Vada for the indicated time points post-inoculation. The data embody arithmetic means (n = 3) plus standard error of the mean. * *p-*value < 0.05; ** *p-*value < 0.01.

The transcript levels of all selected candidate genes are altered in PM attacked epidermis cells compared to the non-inoculated control (**Fig 4**) based on a transcript-profiling Microarray [68].

In the case of CG_1, the detected pattern indicates that the expression is controlled by the circadian clock, while the inoculation with adapted PM fungus *Bgh* as well as the non-adapted fungus *Bgt* led to increased transcript levels. Moreover, elevated transcript levels were observed for CG_2 after inoculation with the non-adapted fungus. Nevertheless, the attack of the adapted fungus led to a transcript peak after 24h, which then declines significantly. The general expression of CG_3 is lower compared to the other candidate genes and the transcript level decreases after *Bgt* inoculation. Furthermore, the transcript of CG_4 is reduced 24h after PM attack. This effect seems to last longer after inoculation with *Bgh* than with *Bgt*.

### Defined alleles vary in the powdery mildew resistance and are in linkage disequilibrium

Regarding the significantly associated SNPs, each selected candidate gene was present in two specific alleles, which differ significantly (*p-*value <0.0001) in their PM resistance (**Table 2**).

**Table 2:** Candidate alleles, their powdery mildew infection values as arithmetic means with standard error based on the infected leaf area from the *Max* BLUEs and their presence (n: Number of genotypes carrying them) in the GWA-panel.

Allele	CG_1	CG_2	CG_3	CG_4
Resistant	22.89 ± 3.28%	24.76 ± 2.48%	28.38 ± 3.48%	24.24 ± 3.09%
	(n = 14)	(n = 21)	(n = 20)	(n = 18)
Susceptible	51.14 ± 0.97%	51.91 ± 0.94%	51.50 ± 0.98%	51.51 ± 0.95%
	(n = 185)	(n = 179)	(n = 179)	(n = 181)

**Fig 5:**
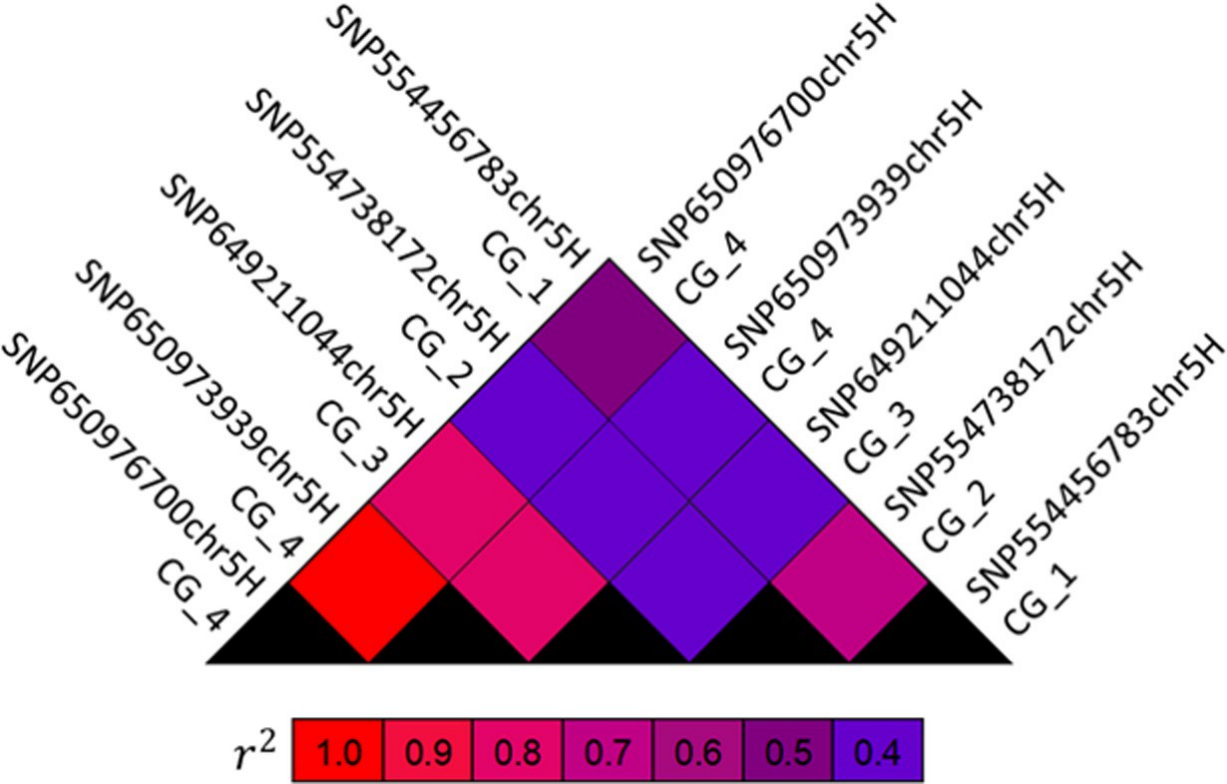
Heatmap-plot of the linkage disequilibrium levels as *r*^2^ values for the Single-Nucleotide Polymorphisms (SNPs) of the selected Candidate Genes (CG). The corresponding *p*-values for each *r*^2^ value were < 0.0001 based on the two-sided Fisher’s exact test.

Additionally, the significant SNPs of the candidates were in LD (*r*^2^ > 0.4), with the highest *r*^2^ observed between the two SNPs of CG_4 (**Fig 5**).

Interestingly, the carriers of the resistant alleles, present in 7-10% of the GWA-panel (**Table 2**), cluster mainly together in a sub-cluster that is defined by Ethiopian landraces (**Fig 2**).

To estimate the effect of the different candidates, a simple linear regression model was fitted on the GWA-panel data for each candidate separately. In addition, a multiple linear regression model including all candidates at once was also fitted. Based on the simple regression models, the candidate genes explain the following proportion of the phenotypic variation: CG_1: 23.4%; CG_2: 31.0%; CG_3: 16.7%; and CG_4: 27.3%. Moreover, the multiple regression model considering all candidates explains only 36.0% of the phenotypic variation.

### Sequence alterations between the selected candidate alleles

For each candidate gene, we defined two alleles; a potential resistant and a susceptible allele (**Table 2**). Since the susceptible alleles originate from Morex, all changes in resistant alleles were according to its sequence (**S1A-S4A Figs in S1 File**). The majority of the annotated and detected SNPs were located in non-coding regions. In the case of CG_1, three synonymous SNPs and one SNP that restores a conserved amino acid (Ile^64^Val) were detected (**S1A Fig in S1 File**). The significant SNP is located at the beginning of the 3’UTR (untranslated region). The protein sequences of both alleles from CG_2 were identical because only one synonymous SNP was determined and the significant SNP is located in the first intron (**S2 Fig in S1 File**). In the resistant allele of CG_3, three synonymous, and eight amino acid exchanges altering the protein sequence were identified (**S3A Fig in S1 File**). Most of these exchanged amino acids were conservative replacements in both, conserved and non-conserved regions. The only exception was a non-conservative amino acid exchange of the conserved Proline^370^ to Leucine (**S3A Fig in S1 File**). The significant SNP is located in the fourth intron. In the case of CG_4, one of the significant SNPs is located in the eleventh intron, while the other one causes an amino acid exchange of the conserved Alanine^373^ to Threonine (**S4 Fig in S1 File**). A new in-frame start codon, introduced by an SNP in the 5’UTR of CG_4 is potentially adding 51 additional amino acids to the N-terminus of the annotated protein (**S4A Fig in S1 File**). Protein sequence analysis by the SignalP server (http://www.cbs.dtu.dk/services/SignalP/) predicts the absence of a signal peptide in both alleles. No further sequence polymorphism was found in the available sequence information, particularly since none of the alleles of this candidate could be recovered as full-length *E*. *coli* clone.

### Initial functional validation of the candidates

**Fig 6:**
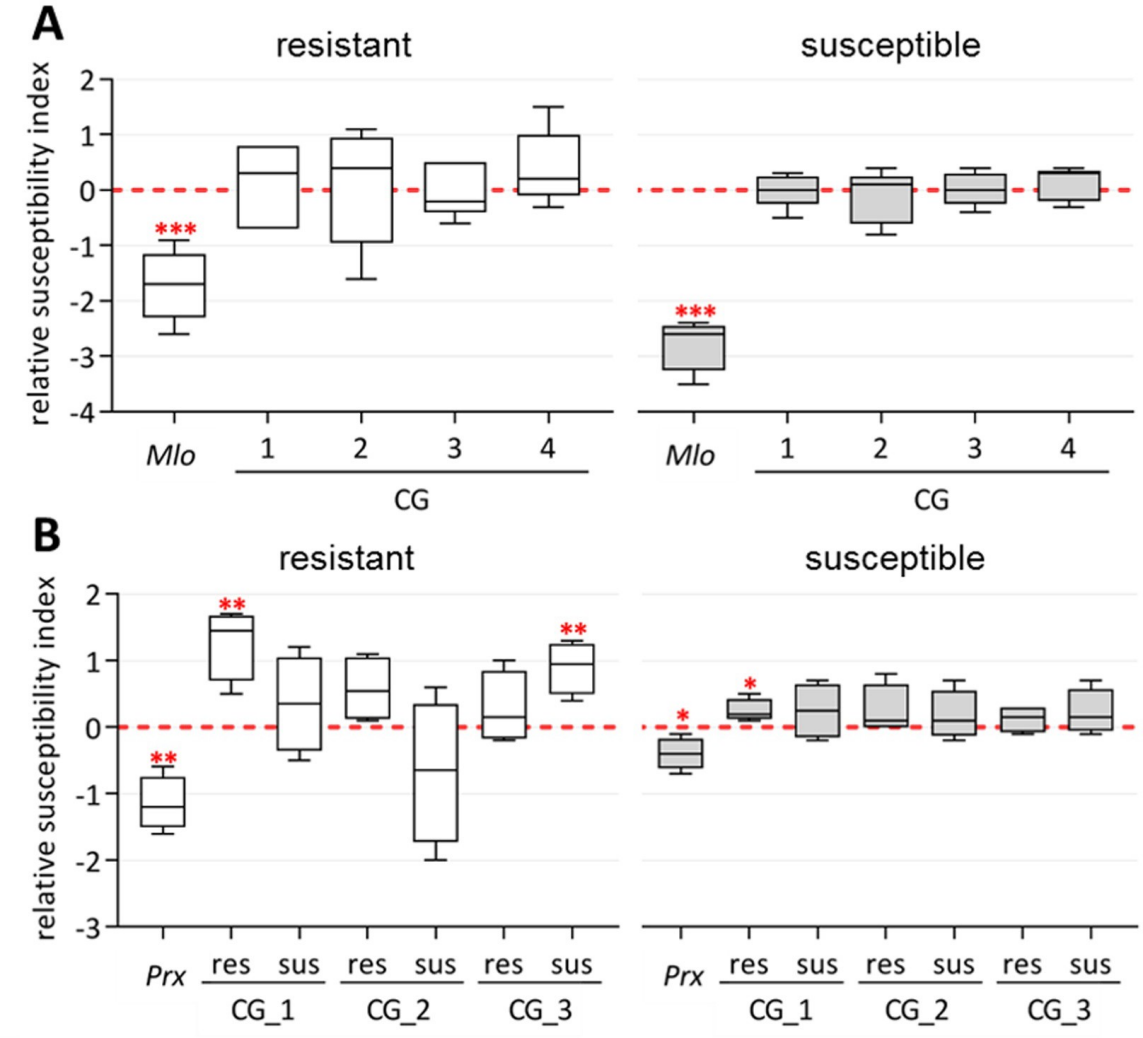
Box plots of the log_2_-transformed relative susceptibility indices of transiently transformed *Hordeum vulgare* epidermis cells. The error bars represent the minimal and maximal values of the susceptibility indices relative to the empty vector control for the transient silencing (n = 5) **(A)** of the indicated candidates (CG) and for the transient overexpression (n = 4) **(B)** in a resistant (WB-052, hollow boxes) and a susceptible barley genotype (Morex, gray-shaded boxes) after inoculation with the *Blumeria graminis* f. sp. *hordei* isolate CH4.8, whereas the putative resistant (res) and the putative susceptible (sus) alleles were tested in the transient overexpression. The corresponding positive controls for the transient silencing: the barley *Mlo* gene (pIPKTA36) and for the transient overexpression: a wheat *Class III peroxidase* (*Prx*, pJP01) are depicted [43,69]. * *p-*value < 0.05; ** *p-*value < 0.01; *** *p-*value < 0.001.

To test the different alleles *in vivo*, leaf segments of a race-specific resistant genotype (WB-052), which carries all resistant alleles, and a susceptible genotype (Morex) carrying the susceptible alleles were bombarded with different constructs. For the TIGS approach, hairpin RNAi constructs targeting the candidate genes were generated and tested [69], however, without a statistically significant effect on the resistance of either genotype (**Fig 6A**).

As mentioned before, obtaining *E*. *coli* clones expressing the full-length coding sequence for GC_4 failed in all attempts. In this regard, only the full-length alleles of CG_1, CG_2, and CG_3 were cloned using genomic DNA to capture also the SNPs located in non-coding regions for the overexpression constructs (**S1-S4 Figs in S1 File**). The overexpression of the resistant allele of CG_1 enhances significantly the susceptibility in terms of an increased relative susceptibility index for both, resistant and susceptible background (**Fig 6B**). Moreover, the overexpression of any of the two alleles of CG_2 did not significantly affect the pathogen resistance in any of the two tested backgrounds. Last but not least, the overexpression of the susceptible allele of CG_3 enhances the susceptibility only in the resistant background.

## Discussion

### Powdery mildew resistance under controlled conditions

Detached leaf assays have been applied for the characterization of race-specific [73–75], as well as for race-nonspecific PM resistance genes [31]. PM isolates used in our study were poly-virulent and displayed overlapping virulence spectra (**S2 Table**), which is reflected in the high correlation between genotype responses against both isolates (**Fig 1**). Only five of the 22 inefficient resistance specificities were specific for one of the isolates. Moreover, cross-talk of defense-related and wound responsive genes has been observed in the past [76] thus, leaves displaying stress or wound responses were excluded from our analyses. The PM response assessed by us on detached seedling leaves under controlled conditions presented high repeatability (0.65–0.93) as well as heritability estimates (0.87–0.88), which evidence the high quality of the data subsequently used for the GWA study.

### Identification of candidate genes in a highly diverse genotype panel

Despite the 28 genotypes with shared geographical origins forming a subpopulation (**Fig 2**and **S1 Table**), only a marginal impact of the population structure is expected because the mean infection of this group did not differ significantly from the rest of the GWA-panel and the correlation between genetic and phenotypic distances is in general low. In contrast, genotypes carrying detected resistant alleles display as a group a significantly reduced mean PM infection (**Table 2**).

In previous studies, genotype panels were restricted to specific geographical regions [30,77], the cultivation status [29,78], or were examined in a candidate gene-based approach [31], thus limiting the genetic diversity being studied. We were able to identify a total of 214 significantly associated SNPs (**S5 Table**) across the three traits (*Max*, RiIII, and D35/3). Furthermore, our approach demonstrates that it is possible to use a highly diverse barley panel to identify candidate genes associated with PM susceptibility.

For functional validation, we focused on two promising association peaks on chromosome 5H (**Fig 3**) co-localizing with the seedling PM resistance locus *Rbgq15* [11]. These last authors proposed the *lipoxygenase2* as the causal gene. Nevertheless, none of the SNPs in the identified corresponding candidate *lipoxygenase* genes (*HORVU5Hr1G093700*, *HORVU5Hr1G093770*, and *HORVU5Hr1G117110*) were significantly associated in our study (**S5 Fig in S1 File**). Furthermore, recent results of Hunt et al. [71] and Gupta et al. [72] identified the same genomic region either as a target region of small RNAs involved in the barley-*Bgh* interaction or as QTL involved in adult plant resistance, respectively. In addition, the potential race-nonspecific resistance gene *HvLSD1b* (LESION SIMULATING DISEASE RESISTANCE 1), i.e. *HvLOL3* (LSD one-like 3; *HORVU5Hr1G093280*) [31], and *Ror2* (*Required for* mlo*-specified resistance*; *HORVU5Hr1G103590*) are located in this genomic region [79]. These data strengthen the importance of the here-identified candidate region of chromosome 5H. Nevertheless, it should be mentioned that the association of SNPs is influenced by several factors like for instance, genetic heterogeneity, and that the most significant markers do not always correspond with causal variants [36,80]. Additionally, it has to be considered that the used exome capture data represent only 1.23% of the barley genome [33]. Besides, the platform design, as well as the candidate gene identification, relied on the annotated genes in the PM susceptible reference genotype Morex, which presumably left additional (candidate) genes carried by the identified PM resistant varieties unexplored [2]. At this point, genotyping with higher coverage as currently done in the BARN project (http://www.eracaps.org/joint-calls/era-caps-funded-projects/) might allow further evaluation of this particular question.

### Selected candidate genes involved in the barley responses to powdery mildew

We identified a total of 214 significantly associated SNPs (**S5 Table**), but for the functional validation, only four candidate genes were selected (**Table 1**) from two promising peaks mapping on chromosome 5H (**Fig 3**). Linkage-mapping studies have shown the influence of loci mapping on the long arm of chromosome 5H on nonhost [7] and adult-plant [72] resistance against PM. Particularly, Gupta et al. [72] reported associated markers that span a 7.6 Mbp region on chromosome 5H and are located between 619.7 and 627.3 Mbp on the reference sequence of Morex. In this respect, our four candidate genes map close (**Table 1**) but are most likely not related to those previously reported QTL (**S5 Fig in S1 File**). Moreover, the two promising association peaks of our study co-localize with a QTL potentially involved in the barley-*Bgh* interaction [11,71]. A very recent transcriptome analysis [81] comparing two barley genotypes with contrasting responses to PM proposed a list of candidate genes controlling the response to PM infection. Interestingly, none of the four candidate genes of our study can be found among their proposed genes, which further highlights the novelty of our findings. Further analysis of significantly associated markers of the present study and the corresponding candidate genes can provide new valuable insights into the barley-*Bgh* plant pathosystem.

Each of the four selected candidate genes explained 17-31% of the phenotypic variation, while this magnitude amounts to 36% for all candidates together. The obtained effect sizes are similar to the reported 6.2–24.8% for *Rbgq15* [11], further supporting the hypothesis that the here presented peaks correspond to this QTL. Only a small increase of the effect size was observed between the different models, which could be related to the LD (**Fig 5**) or a lack of complete additivity between the significant candidate SNPs.

Analyses of race-specific resistance genes indicate specific allelic effects [40,41]. In the present study, two distinct alleles could be annotated for each selected candidate (**S1-S4 Figs in S1 File**), which display a clear effect on the PM resistance (**Table 2**). Also, the transient assays confirm the allelic effects of the candidates (**Fig 6B**). Nevertheless, it has to be considered that the carriers of the potential resistant alleles cluster together (**Fig 2**). In particular, the resistance of WB-052 (HOR2573) might be independent of the here presented candidate genes since it was described as a donor of the *MlLa-H* resistance locus on chromosome 2H [82]. On the other hand, it cannot be ruled out that the observed resistance of WB-052 is caused by the combined effects of the *MlLa-H* resistance specificity and our identified candidate genes.

Because of the moderate LD between the significant candidate SNPs (**Fig 5**), it is possible that the observed PM resistance (**Table 2**) could be related to other (hidden) resistance specificities. Nonetheless, the transcript levels of all four candidate genes were altered in epidermal cells attacked by the adapted (*Bgh*) as well as the non-adapted (*Bgt*) PM fungus (**Fig 4**). These last results further support the involvement of the candidates in the PM responses (**Table 3**).

**Table 3:** Evidence for the candidates to be involved in Powdery Mildew (PM) responses based on the gene function, literature, the observed effect, the expressional regulation after PM attack, and the results of the transient assays.

Candidate	Annotated^a^	Proposed^b^	Literature	Effect^c^	Regulation^d^	TIGS^e^	OX^f^
CG_1	Unknown	AGP	Liang et al. [83]	NEGATIVE	UP	NO	YES
			Wang et al. [84]				
CG_2	DPMT subunit	DPMS3	Jadid et al. [85]	NEGATIVE	UP/DOWN	NO	NO
			Häweker et al. [86]				
CG_3	BTB/POZ/Kelch-associated protein	LRB1/2	Qu et al. [87]	NEGATIVE	DOWN	NO	YES
			Christians et al. [88]				
			Xie et al. [89]				
CG_4	FEN 1-B	FEN 1-B	NO	NEGATIVE	DOWN	NO	NA

^a ^Annotated function: DPMT—dolichol-phosphate-mannosyl-transferase; BTB—Bric-a-Brac/-Tramtrack/-Broad Complex and POZ—POxvirus and Zinc-finger; FEN—flap endonuclease.

^b^ Proposed function based on potential Arabidopsis homologs: AGP–arabinogalactan protein; DPMS3 –dolichol-phosphate-mannose-synthase 3; LRB—light-response BTB.

^c^ Sign of the average difference in infected leaf area between resistant and susceptible alleles.

^d^ Transcript regulation in PM attacked barley epidermis peels.

^e^ Effect of transient silencing.

^f^ Effect of transient overexpression; NA–not analyzed.

To gain further insights into the candidate gene functions, potential homologs of related grasses and model organisms were identified (**S1A-S4A Figs in S1 File**). For CG_1 no function is annotated (**Table 3**) and according to the homologs of *Aegilops tauschii* subsp. *tauschii* and *Brachypodium distachyon*, the candidate belongs to the conserved family of ‘UPF0664 stress-induced protein C29B12.11c’ proteins. Regarding the rice and the Arabidopsis homologs, the candidate (**Table 3**) might be a plasma membrane-bound arabinogalactan protein (AGP) [90,91]. In most homologs, a PH-GRAM-WBP2 (Pleckstrin-Homology-Glucosyltransferases, Rab-like GTPase activators and Myotubularins_WW-binding-protein 2) domain (domain family: cd13214) was annotated. Considering the high sequence identity to our candidate, this domain is conserved (**S1A Fig in S1 File**). In *Magnaporthe oryzae*-inoculated rice, a plasma membrane-bound AGP epitope was specifically upregulated in epidermis cells [83]. Similarly, the transcript level of CG_1 is elevated after PM inoculation (**Fig 4**). Results from Wang et al. [84] further support the hypothesis that CG_1 might function as a circadian clock-regulated defense gene (**Fig 4**). The overexpression of the potential resistant allele leads to enhanced susceptibility in both analyzed backgrounds (**Fig 6B**). This dominant-negative effect on the PM resistance in presence of an active race-specific resistance might be related to the inhibited regulation by the circadian clock, which seems to be naturally maintained even after PM attack (**Fig 4**).

CG_2 is annotated as ‘dolichol-phosphate-mannosyl-transferase subunit’ (**Table 3**) similarly to the annotations of the *A*. *tauschii* and the Arabidopsis homologs (**S2A Fig in S1 File**). In most identified homologs, a dolichol-phosphate-mannosyl-transferase subunit 3 domain is predicted, supporting the hypothesis that the candidate acts as a homolog of the Arabidopsis ‘dolichol-phosphate-mannose-synthase 3’ (DPMS3). DPMS3 is required for the production of isoprenyl-linked glycans [85]. N-glycans are essential for development and immune responses [86,92] and the extensive modification of the plant glycan structure after the fungal attack [93] further supports a candidate function in the response to PM attack. The significant transcript declines after 24h (**Fig 4**) and indicates that the adapted fungus could be specifically able to circumvent innate immune responses. In this respect, it might explain the negative outcome of the transient overexpression (**Fig 6B**). It is possible that *Bgh* could silence the (candidate) transcript similar to a mechanism described in *Botrytis cinerea*, which produces small RNAs perturbing the host immune signaling [94].

The candidate CG_3 and its homologs are annotated as ‘BTB/POZ/Kelch-associated proteins’ (**Table 3**). The ‘Bric-a-Brac/-Tramtrack/-Broad Complex (BTB)/-POxvirus and Zinc-finger (POZ)’ motif is a common protein-protein interaction motif [87]. In Arabidopsis, two protein homologs with high sequence identity (**S3A Fig in S1 File**), firstly described as POZ/BTB Containing-Protein1 and 2 (POB1/2) and later as Light-Response BTB1 and 2 (LRB1/2) [88], were identified. Similar high sequence identity was observed between CG_3 and its potential barley paralog and the two rice orthologs (**S3A Fig in S1 File**). Interestingly, this paralog seems not to be associated with PM resistance. Whether this is a specific candidate effect or caused by genetic heterogeneity [36] needs further investigation. With exception of the nuclear localization signal (NLS), the annotated BTB and BACK (BTB And C-terminal-Kelch) domains were conserved (**S3A Fig in S1 File**), thus supporting the hypothesis that CG_3 is an ortholog of AtLRB1/2. The LRBs constitute Cullin3-based E3 ubiquitin ligases involved in flowering, light-signaling [95], and jasmonate-mediated defense reactions [87]. Particularly, PhyB is essential for light-mediated defense responses [96], while altered resistance responses were described for various *phyB* mutants and pathogens [89,97–99]. The role of CG_3 as a regulator of PhyB in barley needs further investigation. In contrast to the ubiquitous transcription of the *AtLRBs* [88], only low transcript levels were detected in barley and were further reduced after PM attack (**Fig 4**). The enhanced susceptibility observed in the transient overexpression of the potentially susceptible candidate allele (**Fig 6B**) supports this hypothesis; although this effect is only observed in the resistant background (**Fig 6B**). A higher expression of the natural allele may have no additional effect on Morex’s susceptibility. On the other hand, potential dominance effects might mask the effects of the potential resistant candidate alleles, in particular regarding the still active natural allele in the corresponding genotype and the race-specific resistance of WB-052.

CG_4 is annotated as ‘flap endonuclease (FEN) 1-B’ (**Table 3**) and the corresponding barley homolog as FEN1-A (**S4A Fig in S1 File**). A similar protein pair was described in rice. Additional FEN1-B homologs were identified in related grass species (**S4A Fig**), but in Arabidopsis *FEN1* is a single-copy gene [100]. Particularly, the XPG (Xeroderma Pigmentosum Complementation Group G) N-terminal domain and the XPG Internal-region seem to be conserved (**S4A Fig**). In Arabidopsis, FEN1 is essential for genome stability, development, and transcriptional gene silencing [101,102]. Nevertheless, FEN1-B proteins seem to fulfill distinct functions besides their regulatory role in cell proliferation [61]. The *OsFEN1* transcripts are expressed in proliferating tissue and *OsFEN1-B* additionally in mature leaves [61]. Similarly, *FEN1-B* (CG_4) transcripts are detected in mature leaf epidermal cells (**Fig 4**) and are significantly reduced after PM attack. This result supports the candidate involvement in PM responses. Nonetheless, the candidate validation via transient overexpression is pending because the full-length alleles could not be cloned, whereas the transient gene silencing did not lead to significant results for either candidate gene (**Fig 6A**), although the positive control (*Mlo*) indicate the success of the assay. In this context, it has to be considered that this gene has a strong effect on the PM resistance [69].

Altogether, the here presented results give first insights into the functionality of the selected candidate genes and their involvement in race-nonspecific resistance, because: (a) the annotated functions indicate that all four candidates participate in basic (regulatory) processes (**Table 3**); (b) the candidate transcript levels were altered after PM attack of the adapted as well as the non-adapted fungus (**Fig 4**) and (c) the enhanced susceptibility caused by the overexpression of CG_1 (an AGP) and CG_3, the barley homolog of AtLRB1/2 (**Fig 6B**), occurs even after the inoculation with a PM isolate, which was not used for the phenotyping and (d) in the presence of a race-specific resistance background. Furthermore, the results of this work further confirm the complexity of the biological/genetic mechanisms underlying race-nonspecific resistance/susceptibility against fungal plant diseases. At this stage and although promising, these results should be further validated in future research by using other functional techniques such as stable transformants or mutants, before they can have any practical application in barley breeding.

## Supporting information

S1 Table Characterization of the selected 267 barley genotypes (phenotyping panel) for the seedling powdery mildew resistance. (XLSX)

S2 Table Virulence spectra of the powdery mildew isolates CH4.8, D35/3 and RiIII used for the resistance screening on a differential set of 33 barley lines. (XLSX)

S3 Table Used server and databases with the corresponding URLs. (XLSX)

S4 Table Primer sequences used for the generation of the constructs. (XLSX)

S5 Table Significant marker associations with the powdery mildew resistance. (XLSX)

S6 Table Overview of candidate genes including sequence IDs, BLAST and amplification results. (XLSX)

S1 File (DOCX)
