# Supplementary material for: Identification of novel genetic factors underlying the host-pathogen interaction between barley (Hordeum vulgare L.) and powdery mildew (Blumeria graminis f. sp. hordei)
Source: PLoS One. 2020 Jul 2;15(7):e0235565. doi: 10.1371/journal.pone.0235565 (PMC7332009; doi:10.1371/journal.pone.0235565)
Supplement: S1 File — (DOCX) [file pone.0235565.s007.DOCX]

# Supporting Information: Supplementary Figures


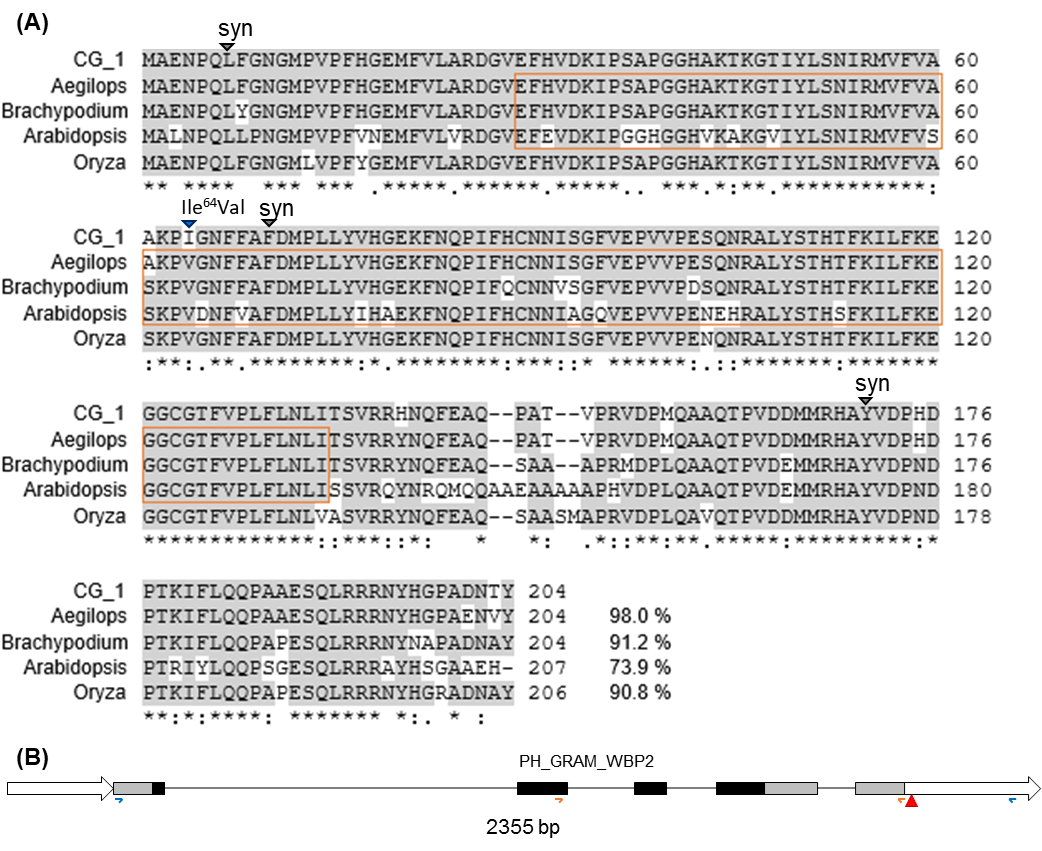


**Figure S1** Multiple sequence alignment of the candidate homologs and gene model of CG_1

**(A)** The complete protein sequences, that were identified as candidate homologs of the indicated plant species, were aligned using the online tool ‘Clustal Omega’ with default settings. The composite alignment underwent minor hand editing and the conserved amino acids were coloured in grey. Dashes (-) denote gaps or missing residues, asterisks (*) represent identical residues, colons (:) indicate conservative substitutions, and dots (.) correspond to similar residues. The predicted PH_GRAM_WBP2 (Pleckstrin Homology-Glucosyltransferases, Rab-like GTPase activators and Myotubularins_WW binding protein 2) domain, that is annotated in the candidate homologs of *Aegilops tauschii*, *Brachypodium distachyon* and Arabidopsis, is indicated by an orange frame. The following sequences were used: candidate *-* HORVU5Hr1G078000; *Aegilops tauschii* subsp. *tauschii* - UPF0664 stress-induced protein C29B12.11c (XP_020176465.1); *Brachypodium distachyon -* UPF0664 stress-induced protein C29B12.11c (XP_003578419.1); *Arabidopsis thaliana* - classical AGP protein (At5g11680.1); *Oryza sativa ssp japonica cv. Nipponbare* - arabinogalactan protein (LOC_Os09g33800.1). The grey coloured triangles indicate the presence of synonymous (syn) amino acid exchanges in the resistant allele compared to the susceptible wild type allele, whereas the blue coloured triangle represents the indicated amino acid exchange. The sequence identity between the candidate gene and the respective homologous sequence are indicated at the end of the alignment.

**(B)** The complete candidate gene model is 2355 bp long. White arrows of the model represent the untranslated regions, boxes the exons and lines the introns. The binding positions of the primers were indicated by coloured arrows (blue: overexpression, orange: silencing), the significant SNP by a red arrow head and the annotated PH_GRAM_WBP2 domain by the black boxes, respectively.


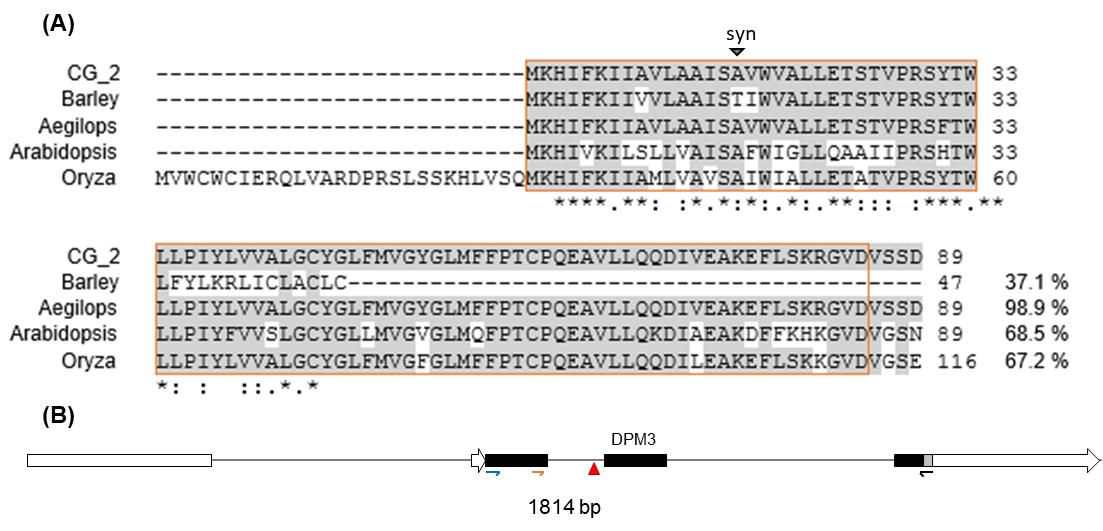


**Figure S2** Multiple sequence alignment of the candidate homologs and gene model of CG_2

**(A)** The complete protein sequences, that were identified as candidate homologs of the indicated plant species, were aligned using the online tool ‘Clustal Omega’ with default settings. The composite alignment underwent minor hand editing and the conserved amino acids were coloured in grey. Dashes (-) denote gaps or missing residues, asterisks (*) represent identical residues, colons (:) indicate conservative substitutions, and dots (.) correspond to similar residues. The DPM3 (dolichol-phosphate mannosyltransferase subunit 3) domain, that is predicted in all protein sequences is indicated by an orange frame. The following sequences were used: candidate - HORVU5Hr1G078330; *Hordeum vulgare* - HORVU3Hr1G017960; *Aegilops tauschii* subsp. *tauschii* - dolichol-phosphate mannosyltransferase subunit 3-like (XP_020176465.1); *Arabidopsis thaliana* - dolichol phosphate mannose synthase 3 (DPMS3) (At1g48140.1); *Oryza sativa ssp japonica cv. Nipponbare* – expressed protein (LOC_Os02g51420.1). The grey coloured triangle indicates the presence of a synonymous (syn) amino acid exchange in the resistant allele compared to the susceptible wild type allele. The sequence identity between the candidate gene and the respective homologous sequence are indicated at the end of the alignment.

**(B)** The complete candidate gene model is 1814 bp long. White arrows of the model represent the untranslated regions, boxes the exons and lines the introns. The binding positions of the primers were indicated by coloured arrows (blue: overexpression, orange: silencing, black: both approaches), the significant SNP by a red arrow head and the annotated DPM3 domain by the black boxes, respectively.


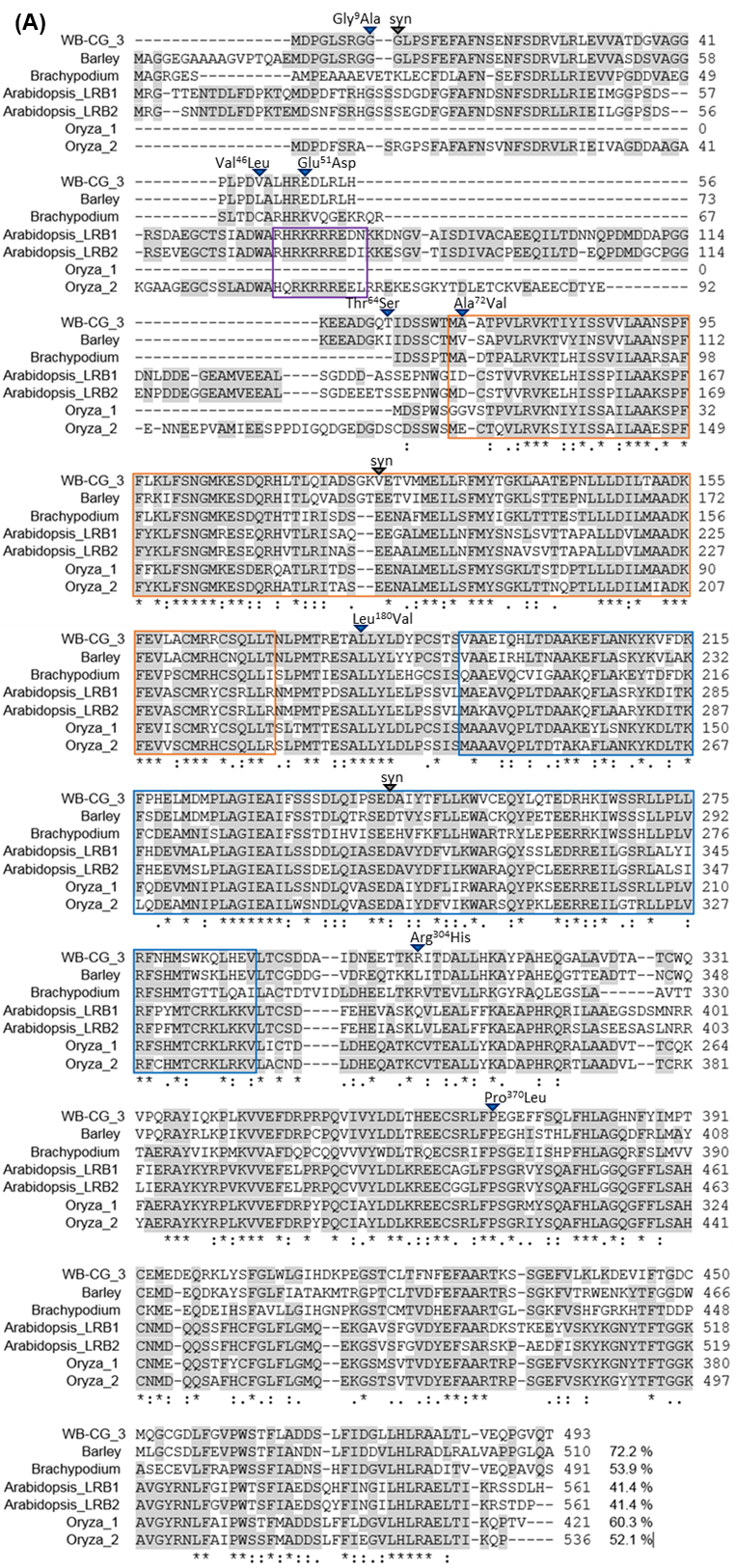


**
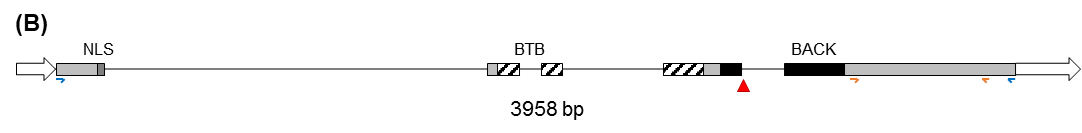
**

**Figure S3** Multiple sequence alignment of the candidate homologs and gene model of CG_3

**(A)** The complete protein sequences, that were identified as candidate homologs of the indicated plant species, were aligned using the online tool ‘Clustal Omega’ with default settings. In case of Arabidopsis, the two homologs were labelled in accordance with the literature (Christians et al., 2012). The composite alignment underwent minor hand editing and the conserved amino acids were coloured in grey. Dashes (-) denote gaps or missing residues, asterisks (*) represent identical residues, colons (:) indicate conservative substitutions, and dots (.) correspond to similar residues. The predicted nuclear localization signals (NLS), the ‘Bric-a-Brac/-Tramtrack/-Broad Complex (BTB) and the BACK (BTB And C-terminal Kelch) domains were indicated by a violet, orange and blue frame, respectively. The following sequences were used: candidate - HORVU5Hr1G116860; *Hordeum vulgare* - HORVU5Hr1G116800; *Brachypodium distachyon* - BTB/POZ domain-containing protein At2g46260 (XP_003559205.2); *Arabidopsis thaliana* LRB1- light-response BTB 1 (AT2G46260.1); *Arabidopsis thaliana* LRB2- light-response BTB 2 (AT3G61600.1); *Oryza sativa ssp japonica cv. Nipponbare* 1 – E1-BTB1 (LOC_Os02g16000.2); *Oryza sativa ssp japonica cv. Nipponbare* 2 – E1-BTB2 (LOC_Os06g31100.1). The grey coloured triangles indicate the presence of synonymous (syn) amino acid exchanges in the resistant allele compared to the susceptible wild type allele, whereas the blue coloured triangles represent the indicated amino acid exchange. The sequence identity between the candidate gene and the respective homologous sequence are indicated at the end of the alignment.

**(B)** The complete candidate gene model is 3958 bp long. White arrows of the model represent the untranslated regions, boxes the exons and lines the introns. The binding positions of the primers were indicated by coloured arrows (blue: overexpression, orange: silencing), the significant SNP by a red arrow head and the annotated domains by the shaded boxes, respectively.


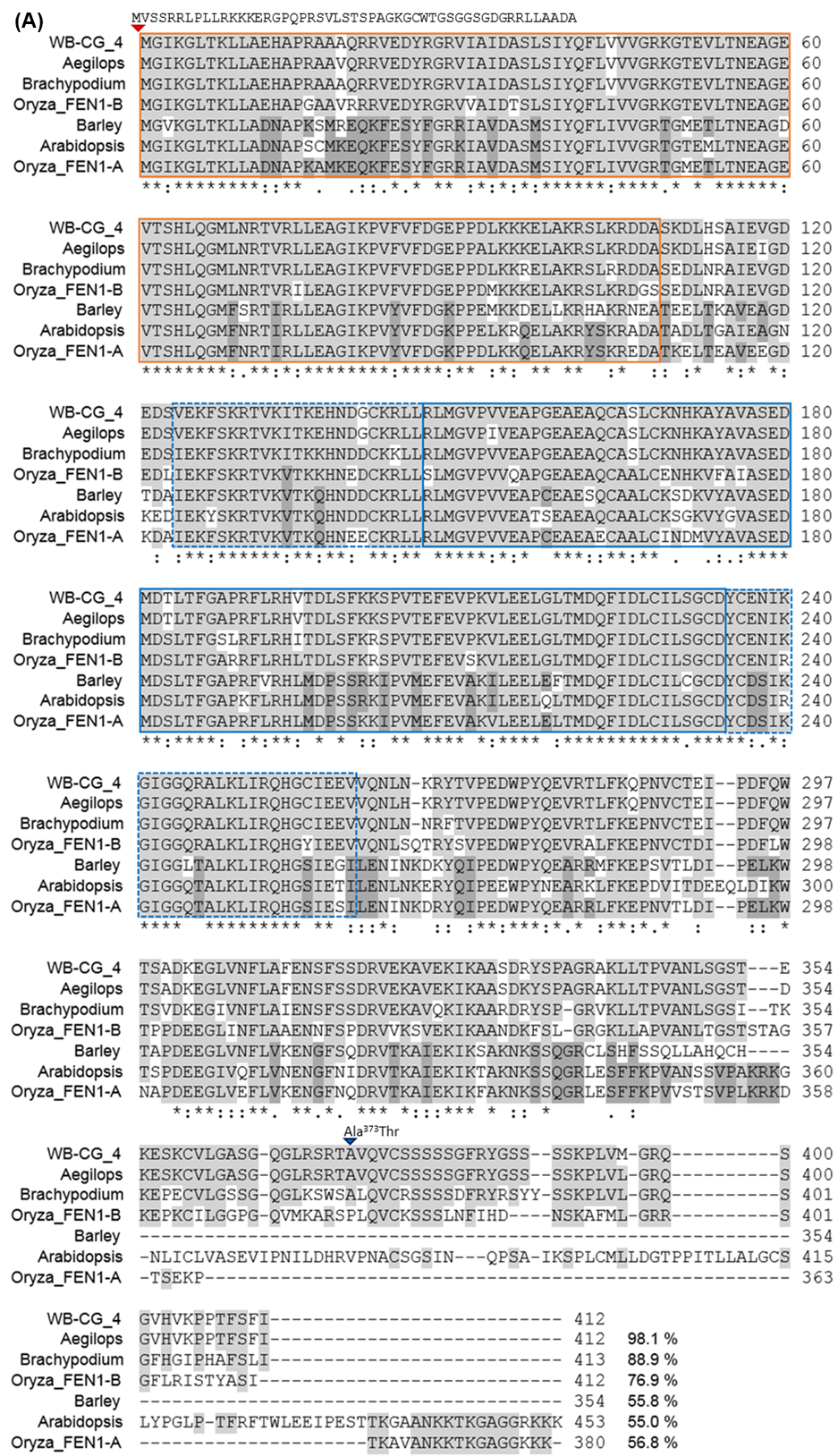


**
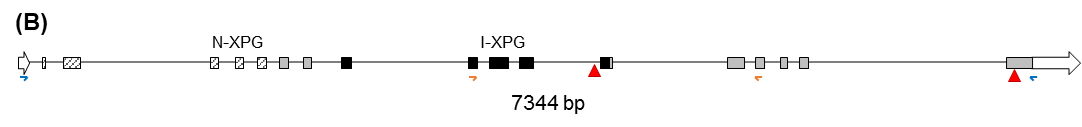
**

**Figure S4** Multiple sequence alignment of the candidate homologs and gene model of CG_4

**(A)** The complete protein sequences, that were identified as candidate homologs of the indicated plant species, were aligned using the online tool ‘Clustal Omega’ with default settings. The composite alignment underwent minor hand editing and the conserved amino acids were coloured in grey. Dashes (-) denote gaps or missing residues, asterisks (*) represent identical residues, colons (:) indicate conservative substitutions, and dots (.) correspond to similar residues. In case of rice, the two homologs were labelled in accordance with the literature (Kimura et al., 2003). The proteins of *Aegilops tauschii*, *Brachypodium distachyon* and WB-CG_4 were annotated as flap endonuclease (FEN)-1 B type proteins and the barley as well as the Arabidopsis homolog as FEN-1 A proteins, respectively. The composite alignment underwent minor hand editing. In case of differences between the conserved amino acids, the FEN-1 A conserved amino acids were coloured dark grey and FEN-1 B amino acids in light grey. Dashes denote gaps. The predicted XPG (Xeroderma Pigmentosum Complementation Group G) N-terminal domain and the XPG Internal-region were indicated by a solid orange and blue frame, respectively. The annotation of the I-domains of the two rice homologs was slightly longer and it is indicated by dashed green frames. The following sequences were used: candidate - HORVU5Hr1G117650; *Aegilops tauschii* subsp. *tauschii* - flap endonuclease 1-B-like (XP_020196748.1); *Brachypodium distachyon* - flap endonuclease 1-B (XP_010239175.2); *Oryza sativa ssp japonica cv. Nipponbare* 1 – flap endonuclease 1-B (XP_015632894.1); *Hordeum vulgare* - HORVU5Hr1G116800; *Arabidopsis thaliana* - flap endo-nuclease I (At5g26680.1); *Oryza sativa ssp japonica cv. Nipponbare* – flap endonuclease 1-A (XP_015639321.1). The blue coloured triangle represents the presence of the indicated amino acid exchange in resistant allele compared to susceptible allele. A mutation in the 5’ untranslated region of the resistant allele led to a new start codon and the respective amino acid sequence is depicted by a red triangle. The sequence identity between the candidate gene and the respective homologous sequence are indicated at the end of the alignment.

**(B)** The complete candidate gene model is 7344 bp long. White arrows of the model represent the untranslated regions, boxes the exons and lines the introns. The binding positions of the primers were indicated by coloured arrows (blue: overexpression, orange: silencing), the significant SNPs by red arrow heads and the annotated domains by the shaded boxes, respectively.


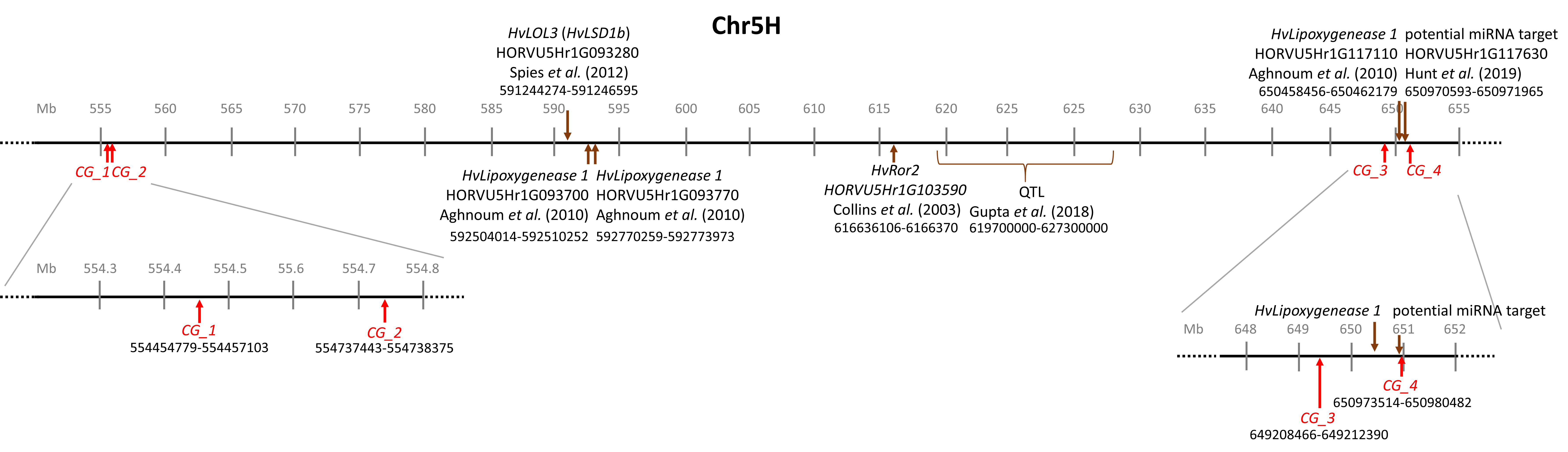


**Figure S5** Schematic representation of the candidate region (555-655 Mb) of chromosome 5H. The candidate genes (CG_1 to CG_4) of the present study are indicated by red arrows. Other interesting genes, which were published in the context of powdery mildew resistance, are labeled by brown arrows or brackets. The numbers under the gene names represent the physical gene positions (in bp) according to the gene models of (Mascher et al., 2017). This candidate region was selected because of the co-localization with the *Rbgq15* locus (Aghnoum et al., 2010). These authors proposed the *lipoxygenase2* as the causal gene of the quantitative trait locus (QTL). Within the candidate region, three potential candidate *lipoxygenase* genes (*HORVU5Hr1G093700*, *HORVU5Hr1G093770*, and *HORVU5Hr1G117110*) were located at the indicated positions. Furthermore, recent results of Hunt et al. (2019) and Gupta et al.(2018) identified the same genomic region as a target region of small RNAs involved in the barley-*Bgh* interaction (*HORVU5Hr1G117630*) and as QTL involved in adult plant resistance, respectively. Additionally, *Ror2* (Required for *mlo*-specified resistance; *HORVU5Hr1G103590*) is also located in this genomic region (Collins et al., 2003). This gene is involved in the penetration resistance against *Bgh* (Freialdenhoven et al., 1996; Collins et al., 2003). The previous GWA study of Spies et al. (2012) identified *HvLSD1b* (SIMULATING DISEASE RESISTANCE 1) respectively *HvLOL3* (LSD one-like 3; *HORVU5Hr1G093280*) as potential race-nonspecific powdery mildew resistance gene.

**Additional Literature**

Aghnoum R, Marcel TC, Johrde A, Pecchioni N, Schweizer P, Niks RE. Basal host resistance of barley to powdery mildew: Connecting quantitative trait loci and candidate genes. Mol Plant-Microbe Interact. 2010;23:91–102. doi: 10.1094/MPMI-23-1-0091.

Christians MJ, Gingerich DJ, Hua Z, Lauer TD, Vierstra RD. The light-response BTB1 and BTB2 proteins assemble nuclear ubiquitin ligases that modify phytochrome B and D signaling in Arabidopsis. Plant Physiol. 2012;160:118–134. doi: 10.1104/pp.112.199109.

Collins NC, Thordal-Christensen H, Lipka V, Bau S, Kombrink E, Qiu JL, et al. SNARE-protein-mediated disease resistance at the plant cell wall. Nature. 2003;425:973–7. doi: 10.1038/nature02076.

Freialdenhoven A, Peterhansel C, Kurth J, Kreuzaler F, Schulze-Lefert P. ldentification of Genes Required for the Function of Non-Race-Specific. 1996. *The Plant Cell*, *8*, 5–14. doi: 10.1105/tpc.8.1.5.

Gupta S, Vassos E, Sznajder B, Fox R, Khoo KHP, Loughman R, et al. A locus on barley chromosome 5H affects adult plant resistance to powdery mildew. Mol Breed. 2018;38:103. doi: 10.1007/s11032-018-0858-2.73. Dreiseitl A. Heterogeneity of powdery mildew resistance revealed in accessions of the ICARDA wild barley collection. Front Plant Sci. 2017;8:202. doi: 10.3389/fpls.2017.00202.

Hunt M, Banerjee S, Surana P, Liu M, Fuerst G, Mathioni S, et al. Correction to: small RNA discovery in the interaction between barley and the powdery mildew pathogen. BMC Genomics. 2019;20:697. doi: 10.1186/s12864-019-6012-7.

Kimura S, Furukawa T, Kasai N, Mori Y, Kitamoto HK, Sugawara F, et al. Functional characterization of two flap endonuclease-1 homologues in rice. Gene. 2003;314:63–71. doi: 10.1016/s0378-1119(03)00694-2.

Mascher M, Gundlach H, Himmelbach A, Beier S, Twardziok SO, Wicker T, et al. A chromosome conformation capture ordered sequence of the barley genome. Nature. 2017;544:427–433. doi: 10.1038/nature22043.

Spies A, Korzun V, Bayles R, Rajaraman J, Himmelbach A, Hedley PE, et al. Allele mining in barley genetic resources reveals genes of race-non-specific powdery mildew resistance. Front Plant Sci. 2012;2:113. doi: 10.3389/fpls.2011.00113.
